# A case control investigation of COVID-19 associated mucormycosis in India

**DOI:** 10.1186/s12879-022-07844-y

**Published:** 2022-11-16

**Authors:** Tanu Anand, Aparna Mukherjee, Aanchal Satija, Poonam Sharma Velamuri, Kh. Jitenkumar Singh, Madhuchhanda Das, Kripa Josten, Pragya D. Yadav, Rima R. Sahay, Archana Y. Keche, Nitin M. Nagarkar, Prashant Gupta, D. Himanshu, Sejal N. Mistry, Jimy D. Patel, Prajwal Rao, Shalesh Rohatgi, Soumitra Ghosh, Avijit Hazra, Anupma Jyoti Kindo, Radha Annamalai, Shivaprakash M. Rudramurthy, Mini P. Singh, Mohammad Shameem, Nazish Fatima, Janakkumar R. Khambholja, Sangita Parikh, Manisha Madkaikar, Vandana D. Pradhan, Sushila Kataria, Pooja Sharma, Samiran Panda, ICMR-Mucormycosis group, Anita M. Shete, Triparna Majumdar, Priya Abraham, Anudita Bhargava, Rupa Mehata, Ripu Daman Arora, Richa Tigga, Gopa Banerjee, Vijay Sonkar, H. S. Malhotra, Neeraj Kumar, Rajashri Patil, Chandrashekhar G. Raut, Kumkum Bhattacharyya, Preetam Arthur, L. Somu, Padma Srikanth, Naresh K. Panda, Dipti Sharma, Wasil Hasan, Aftab Ahmed, Meeta Bathla, Sunita Solanki, Hiren Doshi, Yash Kanani, Nishi Patel, Zincal Shah, Alok Kumar Tembhurne, Chhaya Rajguru (Waghmare), Lalitkumar R. Sankhe, Shrinivas S. Chavan, Reetika Malik Yadav, Vikas Deswal, Kuldeep Kumar

**Affiliations:** 1 Indian Council of Medical Research, New Delhi, India; 2 ICMR-National Institute of Medical Statistics, New Delhi, India; 3 ICMR-National Institute of Virology, Pune, India; 4 All India Institute of Medical Sciences, Raipur, India; 5 King George’s Medical University, Lucknow, India; 6 Pandit Dindayal Upadhyay Medical College, Rajkot, India; 7 Dr. D. Y. Patil Medical College, Hospital and Research Centre, Pune, India; 8 Institute of Post Graduate Medical Education and Research, Kolkata, India; 9 Sri Ramachandra Medical College and Research Institute, Chennai, India; 10 Post Graduate Institute of Medical Education and Research, Chandigarh, India; 11 Jawaharlal Nehru Medical College Aligarh Muslim University, Aligarh, India; 12 Smt. NHL Municipal Medical College, Ahmedabad, India; 13 AMC MET Medical College, Ahmedabad, India; 14 ICMR-NIIH, Mumbai, India; 15 Medanta-The Medicity, Gurugram, India

**Keywords:** Mucormycosis, Satellite, Epidemic, Corticosteroids, Diabetes

## Abstract

**Background:**

Increased occurrence of mucormycosis during the second wave of COVID-19 pandemic in early 2021 in India prompted us to undertake a multi-site case–control investigation. The objectives were to examine the monthly trend of COVID-19 Associated Mucormycosis (CAM) cases among in-patients and to identify factors associated with development of CAM.

**Methods:**

Eleven study sites were involved across India; archived records since 1st January 2021 till 30th September 2021 were used for trend analysis. The cases and controls were enrolled during 15th June 2021 to 30th September 2021. Data were collected using a semi-structured questionnaire. Among 1211 enrolled participants, 336 were CAM cases and 875 were COVID-19 positive non-mucormycosis controls.

**Results:**

CAM-case admissions reached their peak in May 2021 like a satellite epidemic after a month of in-patient admission peak recorded due to COVID-19. The odds of developing CAM increased with the history of working in a dusty environment (adjusted odds ratio; aOR 3.24, 95% CI 1.34, 7.82), diabetes mellitus (aOR: 31.83, 95% CI 13.96, 72.63), longer duration of hospital stay (aOR: 1.06, 95% CI 1.02, 1.11) and use of methylprednisolone (aOR: 2.71, 95% CI 1.37, 5.37) following adjustment for age, gender, occupation, education, type of houses used for living, requirement of ventilatory support and route of steroid administration. Higher proportion of CAM cases required supplemental oxygen compared to the controls; use of non-rebreather mask (NRBM) was associated as a protective factor against mucormycosis compared to face masks (aOR: 0.18, 95% CI 0.08, 0.41). Genomic sequencing of archived respiratory samples revealed similar occurrences of Delta and Delta derivates of SARS-CoV-2 infection in both cases and controls.

**Conclusions:**

Appropriate management of hyperglycemia, judicious use of steroids and use of NRBM during oxygen supplementation among COVID-19 patients have the potential to reduce the risk of occurrence of mucormycosis. Avoiding exposure to dusty environment would add to such prevention efforts.

**Supplementary Information:**

The online version contains supplementary material available at 10.1186/s12879-022-07844-y.

## Introduction

The COVID-19 pandemic has adversely affected the world. As on 08th July 2022, globally, 558,703,551 confirmed cases and 6,369,057 deaths due to COVID-19 were reported [1]. The COVID-19 illness is further compounded by co-occurrence of fungal infections among patients with COVID-19, weeks or months after their recovery. Such occurrences of COVID-19 Associated Mucormycosis (CAM) were reported from several countries [2]. The unprecedented increase in cases of CAM in India during the second wave of the pandemic in May 2021 became a cause of concern and strained the already overwhelmed healthcare system [3]. By the end June 2021, 40,824 cases of mucormycosis had been reported from India with 3229 patients succumbing to death [4].

Mucormycosis is an opportunistic fungal infection caused by *Mucorales*. It is an invasive disease with protracted clinical course, challenging treatment options and a very high mortality rate, which has increased from 41% in pre-COVID-19 era to 49% during the pandemic [3, 5, 6]. Several equivocal hypotheses have been put forth regarding the risk factors associated with development of CAM. Systematic reviews from India and elsewhere have identified factors such as low oxygen milieu, diabetes mellitus (DM), inappropriate doses and duration of glucocorticoid use, host innate immunity related issues and prolonged duration of hospital stay with or without mechanical ventilation to be responsible for development of mucormycosis among COVID-19 patients [7]. A retrospective study from India before the second wave of the pandemic, during September-December 2020, revealed COVID-19 related hypoxemia and improper glucocorticoid use to be associated with CAM [8]. However, the surge of CAM cases during the second wave of the pandemic in India from May 2021 onward underlined the need for further in-depth investigation. Against this background, we conducted a multi-site case control investigation with the objectives of examining the monthly trend of proportion of CAM cases among in-patients since 1st January 2021 and identifying factors associated with the development of CAM post-second wave.

## Material and methods

### Study settings and participants

Study sites were the hospitals selected from the National Clinical Registry for COVID-19 as well as institutions associated with the Indian Council of Medical Research (ICMR) mycology research network [9]. Eleven sites were chosen across the country and grouped under four zones based on their geographic locations, namely: North, East, West and South plus Central. The sites were shortlisted based on a selection matrix, which included willingness to conduct the study, infrastructure for COVID-19 testing, capacity for diagnosis of fungal diseases and investigators’ research expertise. Each research team consisted of microbiologists and clinicians such as ophthalmologists, general physicians, otolaryngologists, and radiologists.

### Trend analysis

Medical record-based analysis of monthly trend of confirmed cases of CAM was carried out in the participating hospitals during 1st January 2021 to 30th September 2021. The total number of patients admitted in the same hospitals were considered as denominator.

### Cases and controls

A case was considered as CAM when a COVID-19 patient (active or recovered, any age and gender) was suspected of mucormycosis clinically and confirmed microbiologically for the same [10]. All specimens (either biopsy from paranasal sinus or nasal discharge or broncho-alveolar lavage or sputum) were considered microbiologically positive if aseptate or sparsely septate broad ribbon-like hyphae, with 90° branching angles were observed under direct microscopy in wet mount with potassium hydroxide (KOH) or lactophenol cotton blue or Periodic Acid Schiff (PAS) or Grocott-Gomori’s Methenamine-Silver (GMS) stain and/or culture on Sabouraud Dextrose Agar (SDA) showed cottony rapid growth with or without black heads, followed by identification of fungus by microscopy. Two controls were enrolled from a population of all COVID-19 patients discharged from the same hospital, 3–4 weeks prior to the date of diagnosis of an enrolled case of mucormycosis.

Suspected cases of mucormycosis, not microbiologically confirmed, irrespective of the treatment received and critically ill patients, unable to participate in the interview, were excluded from the study. The cases and controls were enrolled during 15th June 2021 to 30th September 2021. An individual once enrolled as control, was not considered in the population of controls for subsequent cases.

### Sample size and sampling

Based on previous reports, the least common risk factor for CAM was intensive care unit (ICU) stay. Taking the proportion of CAM and COVID-19 in-patients requiring ICU stay as 68% and 30%, respectively [11, 12] and a conservative Odds Ratio (OR) of 3, the sample size was estimated using Open Epi v3.0 (Rollins School of Public Health, Emory University) for unmatched case control design [13]. With an allocation ratio of 1:2, the sample size was calculated as 73 cases and 145 controls at the power of 95% and alpha error of 5%. In each zone (North, East, West and South plus Central), 218 patients (73 cases of CAM and 145 controls of COVID-19 without mucormycosis) were attempted to be enrolled with overall sample size of 872. Due to the dynamic situation of the pandemic and differential burden of disease during the study period, there was unequal enrolment from different geographic locations. The zone wise enrolment of cases and controls is depicted in the spot map presented as Additional file 1: Fig S1.

### Study tools and data collection

Data were collected using a structured questionnaire with the following domains: (i) socio-demographic profile, (ii) present hospitalization, (iii) past hospitalization (in last 3 months), and (iv) home-based care for COVID-19. Data were extracted from the medical records of eligible cases and controls (as described above) by designated project staff posted at each of the study sites. They were trained by using virtual platform on how to use the paper-based clinical investigation reporting form (CRF) prior to data collection. The cases were enrolled while they were admitted for treatment at the hospital while the participants selected as controls were contacted telephonically. Their contact details and other pertinent clinical information were retrieved from hospital records. Confidentiality of the consenting participants was maintained, and patient identifiers were not entered in the CRF. They were requested to share their laboratory reports and treatment records on WhatsApp or via electronic mail (if not available from the hospital records).

### Detection of variants through next generation sequencing

A subset of naso and oro-pharyngeal swab samples were transported to the ICMR-National Institute of Virology (ICMR-NIV), Pune in dry ice for next generation sequencing (NGS). Total RNA was extracted from 400 µl of each specimen using the Magmax™ Viral RNA/Pathogen RNA Extraction kit (Applied Biosystems, ThermoScientific, USA). Specimens with E gene Ct < 30 were processed for SARS-CoV-2 whole genome sequencing using the amplicon-based COVID Seq method (Illumina, USA) as per the instructions of the manufacturer. Total RNA was also tested for N and E subgenomic RNA (sgRNA). Viral gRNA and sgRNA were calculated using standard curve as described earlier [14]. NGS was performed using the Covidseq kits as described earlier [15].

### Data management and analyses

Information collected on paper CRFs were entered in web portal designed by ICMR with unique login IDs for each participating site. Collation, validation, cleaning, and verification were carried out at the ICMR Headquarters. Chi-square test was used for trend analysis. Descriptive analyses of socio-demographic, clinical and laboratory profiles of all cases and controls were undertaken. Statistical analyses were performed using student ‘t’ or Mann Whitney U test as appropriate for continuous variables while categorical variables were compared using Chi-square or Fisher’s exact test. Probability at 5% level was considered as statistically significant. Multivariable logistic regression was used to identify factors associated with the development of CAM. Variables with significance at p value < 0.05 in bivariate analysis were included in logistic regression model. Doses of steroids such as dexamethasone, methylprednisolone and hydrocortisone were converted to equivalent doses of prednisolone and multiplied with the duration to obtain the administered cumulative dose. All statistical analyses were performed using R CRAN (version 4.0.2) software.

### Ethical considerations

Approvals were obtained from the ICMR-Central Ethics Committee on Human Research and the Institutional Ethics Committee of each of the participating sites. Written informed consent was obtained from CAM cases while controls were contacted telephonically, and verbal consent was taken and recorded in the consent form by the interviewer. Parental consent was obtained for children aged less than 18 years. Verbal and written assent of child were obtained for children between 7 to 12 years and those above 12, respectively.

## Results

### Trend analysis of CAM cases and controls

**Fig. 1:**
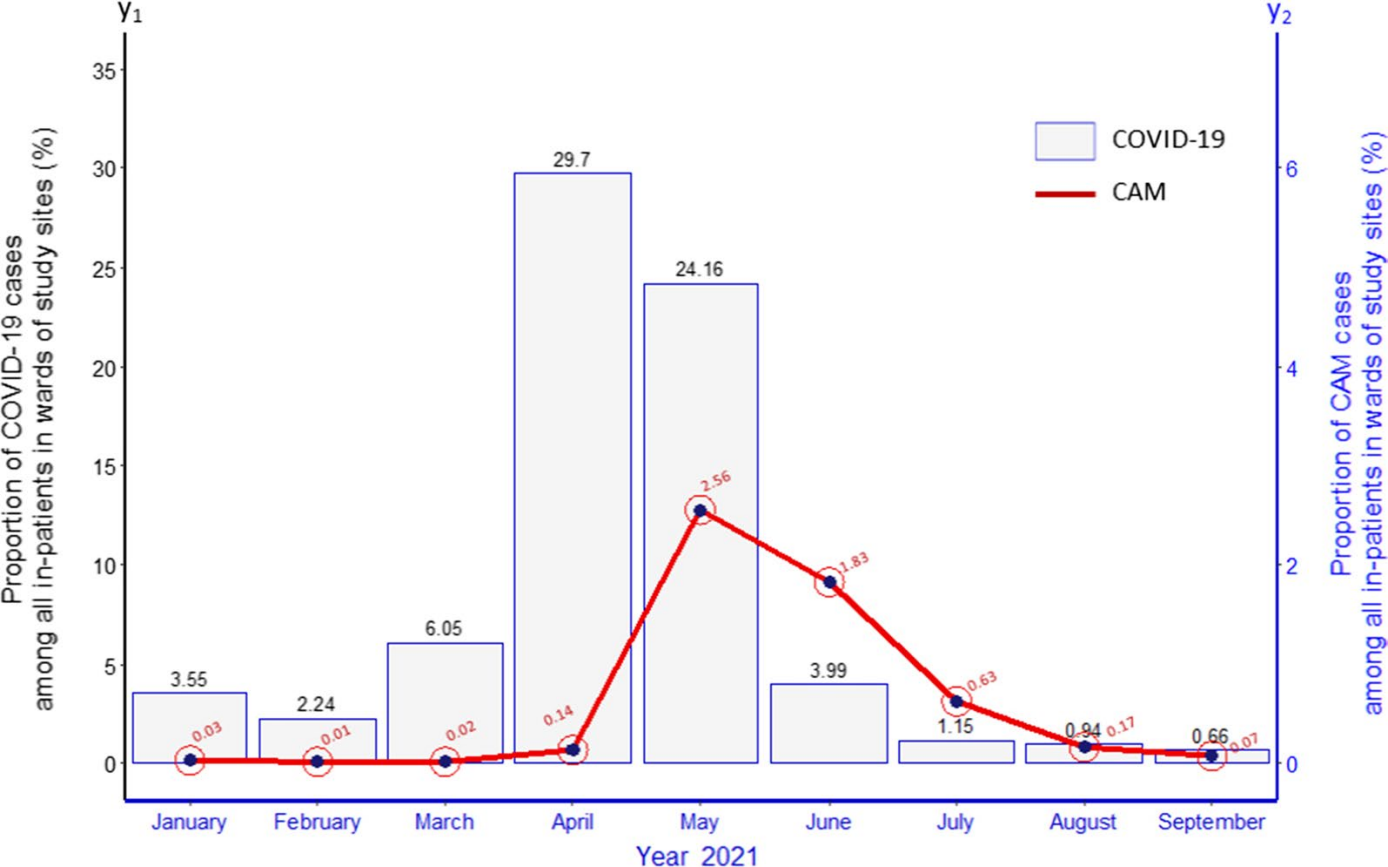
Monthly trends for COVID associated mucormycosis (CAM) and COVID-19 cases

Analysis of monthly trends revealed that CAM cases reached their peak during the month of May 2021 and then declined gradually till September 2021. The trend had an overall χ^2^ value of 5189.7 (p < 0.001). There was a lag of one month between the peak of COVID-19 cases admitted in hospitals and satellite peak of CAM cases as evident in Fig. 1.

### Socio-demographic and clinical characteristics of CAM cases and controls

**Fig. 2:**
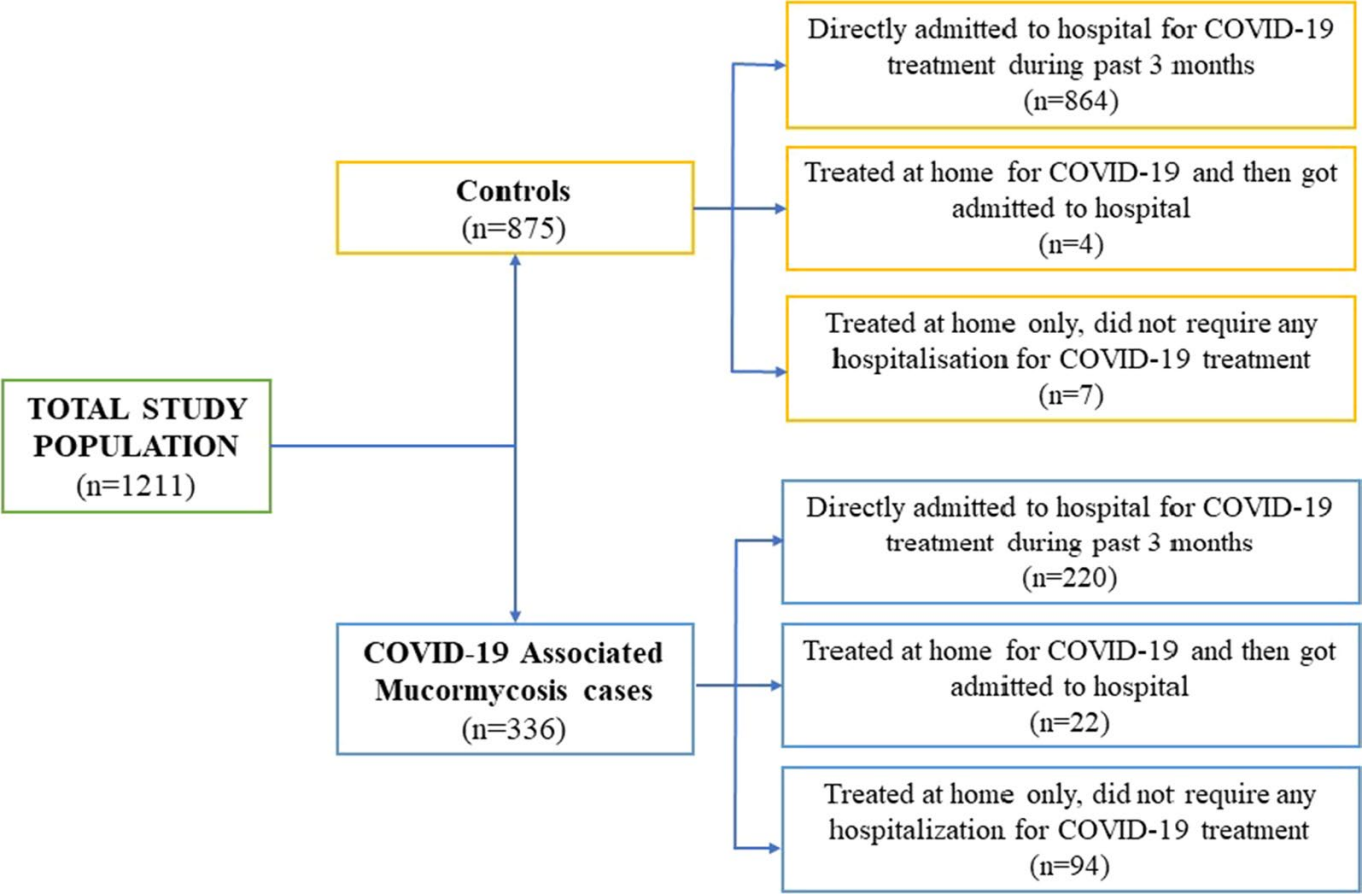
Study population enrollment flowchart

Of the 1235 patients screened for participation in this study from 11 sites, patients from one study site (n = 15) were excluded from analyses as they did not adhere to the criteria for case definition. Three more patients were excluded as microbiological confirmation of mucormycosis was not available and six were excluded because of non-availability of COVID-19 history. Thus, 1211 patients were enrolled in the study from 10 sites; 336 (27.7%) were CAM cases and 875 (72.3%) were COVID-19 positive non-mucormycosis controls. The study population was representative of four zones of the country namely, North (CAM: 24, 7.2%, COVID-19: 47, 5.4%), East (CAM: 30, 8.9%, COVID-19: 65, 7.4%), West (CAM: 119, 35.4%, COVID-19: 452, 51.7%), South plus Central (CAM: 163, 48.5%, COVID-19: 311, 35.5%). The details of the study enrollment are depicted in Fig. 2 and presented as a spot map in Additional file 1: Fig. S1.

The socio-demographic characteristics of cases and controls are depicted in Table 1. Nearly half of the CAM cases (157/336; 46.7%) belonged to 45 to 59-year age group, while 31.2% of the controls were in this age bracket. A significantly higher proportion of CAM cases were males (232/336; 69.1%). Most of the CAM and controls had studied till 12th standard (CAM: 185/336, 55.1%; Controls: 473/875, 54.1%). As compared to COVID-19 controls, a significantly higher proportion of CAM cases worked in dusty environment in daily life, being involved in either farming or gardening or both (n = 94, 27.9%) or working at construction sites (n = 19, 5.7%). A significantly higher proportion of CAM cases lived in cemented houses with thatched/asbestos roof or mud houses with thatched roof as compared to controls. The median (IQR) interval between COVID diagnosis and admission due to mucormycosis was 31 days (18, 47). The most frequent symptoms reported by CAM cases at the time of hospitalization due to mucormycosis were oro-facial symptoms namely toothache, facial swelling, or pain (n = 260, 77.4%) and ophthalmological symptoms like swelling, pain or redness in eye, blurry or double vision (n = 210, 62.5%) followed by lesser frequent symptoms such as headache, weakness, fever, cough, breathlessness, gastrointestinal or neurological symptoms. Symptom frequency of CAM cases at the time of hospitalization due to mucormycosis is presented in Additional file 2: Fig. S2.

**Table 1:** Socio-demographic characteristics of cases and controls

Variables	Mucormycosis cases (n = 336)	Controls (n = 875)	p value^#^
Age categories (in years)			
Less than 44	110 (32.7)	375 (42.9)	
45 to 59	157 (46.7)	273 (31.2)	< 0.001
60 & above	69 (20.6)	227 (25.9)	
Gender			
Male	232 (69.1)	535 (61.1)	0.011
Female	104 (30.9)	340 (38.9)	
Occupation			
Unemployed	112 (33.3)	349 (39.9)	0.03
Employed	224 (66.7)	526 (60.1)	
Education			
Illiterate	75 (22.3)	114 (13.0)	
Till 12th Standard	185 (55.1)	473 (54.1)	< 0.001
Graduate	60 (17.9)	224 (25.6)	
Post-graduate	16 (4.7)	64 (7.3)	
Daily life work in dusty environment			
No	223 (66.4)	704 (80.5)	
Farming/gardening	94 (27.9)	129 (14.7)	< 0.001
Construction sites	19 (5.7)	42 (4.8)	
Type of house			
Cemented house	227 (67.6)	690 (78.8)	
Cemented house with thatched roof	55 (16.4)	76 (8.7)	< 0.001
Cemented house with asbestos roof	25 (7.4)	47 (5.4)	
Mud house with thatched roof	29 (8.6)	62 (7.1)	

^#^χ^2^ test performed for analysis

Values are expressed as n (%)

### Next generation sequencing and phylogenetic analysis

**Fig. 3:**
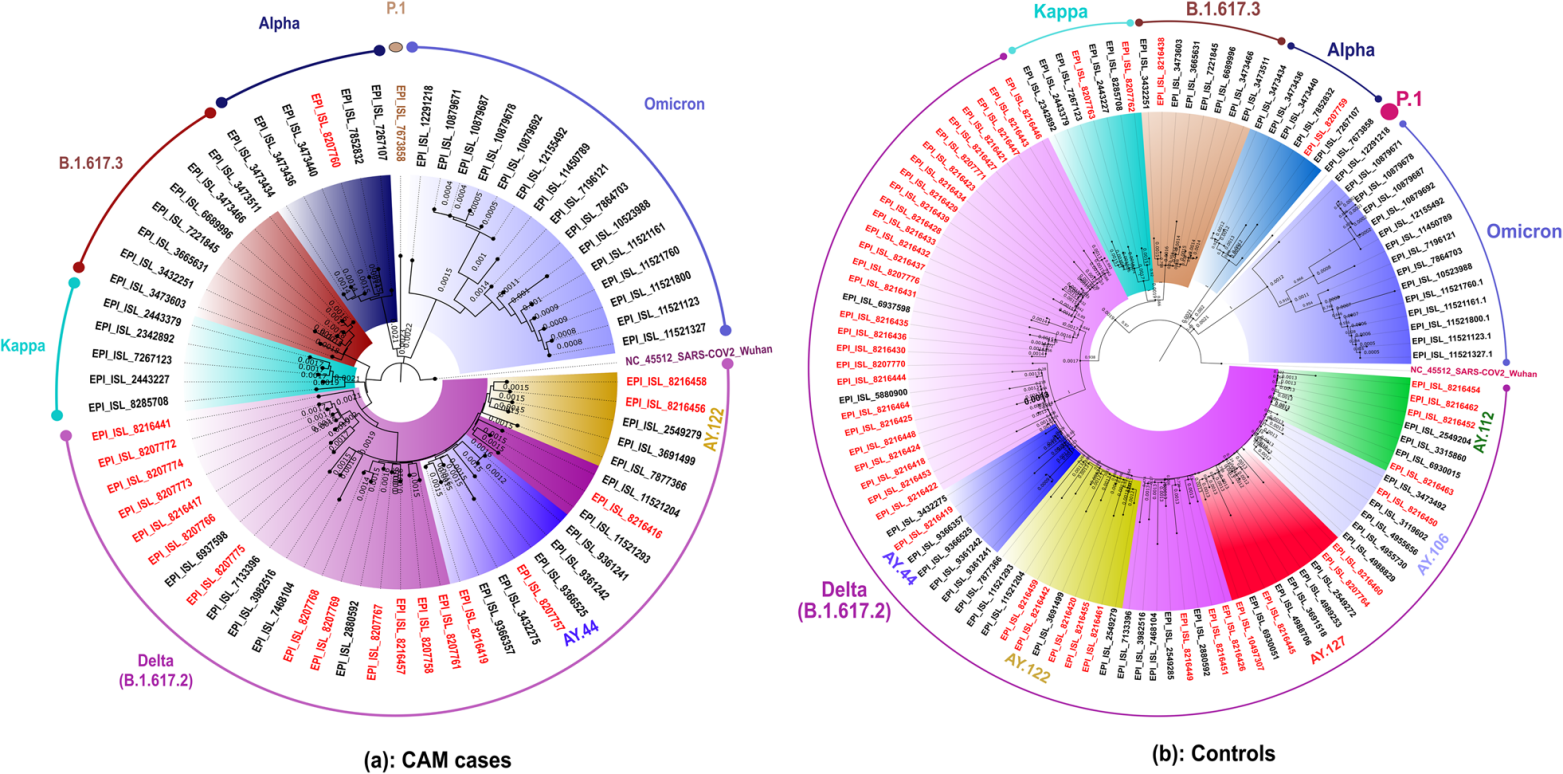
Maximum likelihood tree for the SARS-CoV-2 genomes retrieved in the study*. *Representative SARS-CoV-2 sequences from different lineages along with 81 sequences retrieved were used to generate the tree with a bootstrap replication of 1000 cycles. The retrieved pangolin lineages are marked on branches in different colors. The accession numbers of the retrieved sequences are highlighted in red. Fig Tree v1.4.4 and Inkscape were used to visualize and edit the generated tree

Next generation sequencing was performed on the samples from cases and controls having E gene Ct value < 30; samples from 29/43 (64.4%) CAM cases and 52/71 (73.2%) controls were sequenced. Complete genome could be retrieved from 19/29 (65.5%) CAM cases and 50/52 (96.2%) controls sequenced. The pangolin lineage for the genomic sequences with more than 98% retrieval was obtained [20 cases and 49 controls] using the online website (https://cov-lineages.org/resources/pangolin.html). Predominant SARS-CoV-2 lineages detected were B.1.617.2 [Delta variant] (CAM: n = 14 vs. Controls: n = 34), followed by AY.122 (CAM: n = 2 vs. Controls: n = 5) and AY.112 (CAM: n = 1 vs. Controls: n = 3). Frequencies of AY.44 and B.1.1.7 (Alpha variant) were equal in both CAM and controls (CAM: n = 1 vs. Controls: n = 1). Absence of AY.106, B.1.617.3 and AY.127, B.1.617.1 (Kappa variant) was observed in CAM cases. However, one sample each for former two and two samples each for latter two were observed in the control group. Details of the genomic reads mapped, total reads, pangolin lineage and the accession numbers for each of the strains retrieved are presented in Additional file 3: Table S1. A phylogenetic tree depicts a clear segregation of the clades (Fig. 3). The amino acid variation observed in the spike gene regions is depicted in Additional file 4: Fig. S3; this figure indicates the presence of similar mutations across the CAM cases and controls.

### Clinical and management profile of participants treated at hospitals due to COVID-19

The clinical, laboratory characteristics and management profile of the CAM cases were compared with that of controls as presented in Table 2. A higher proportion of CAM cases had headache (83; 36.7%) and central nervous system (CNS) symptoms (20; 8.8%) during their COVID-19 illness as compared to controls. Cases stayed for longer duration in hospitals [CAM: 9 days (6,12) vs. Controls: 7 days (4, 10), p < 0.001]. The COVID-19 patients who went on to develop CAM had a higher proportion of DM [(212; 87.6%) vs. (156; 17.9%)] as well as poorly controlled DM as evident through higher mean HbA1c (7.0 ± 2.8 vs. 5.9 ± 2.2, p < 0.001) in them. Participants who developed CAM later had a higher median level of random blood sugar at admission as well as higher maximum blood sugar level measured during hospital stay. It was intriguing that only a few patients of CAM had history of illnesses such as chronic obstructive pulmonary disease (n = 2, 0.2%) or cancer (4, 0.4%). Oxygen requirement was significantly higher in CAM cases as compared to COVID-19 controls [(144; 59.5%) vs. (446; 51.4%)]. The comparison of oxygen and steroid usage between CAM cases and controls are presented in Table 2.

**Table 2:** COVID-19 clinical, laboratory and treatment profile of in-patient participants

Variables		Mucormycosis cases (n = 242)*	Controls (n = 868)	p value
Symptomatology^#^		*n* = *226*	*n* = *807*	
Fever		180 (79.6)	640 (79.3)	0.91^##^
Cough		131 (57.9)	535 (66.3)	0.02^##^
Breathlessness		94 (41.6)	369 (45.7)	0.27^##^
Headache		83 (36.7)	187 (23.2)	< 0.001^##^
Generalized weakness		92 (40.7)	345 (42.8)	0.58^##^
Loss of taste/smell		48 (21.2)	128 (15.9)	0.06^##^
Gastrointestinal symptoms		24 (10.6)	70 (8.7)	0.37^##^
Otorhinolaryngological symptoms		40 (17.7)	174 (21.6)	0.21^##^
Central nervous system symptoms		20 (8.8)	28 (3.5)	0.001^##^
Duration of hospital stay in days, median (IQR)		9 (6,12)	7 (4, 10)	< 0.001^**^
Days in^#^, median (IQR)				
Ward		8 (6, 12)	7 (4, 10)	< 0.001^**^
Cabin/private ward		13.5 (7.5, 18)	8 (4, 11)	0.15^**^
High Dependency Units		5 (4, 8)	7 (5, 10)	0.27^**^
Intensive Care Unit		7 (5,11)	7 (4, 12)	0.77^**^
Underlying medical conditions				
Diabetes mellitus		212 (87.6)	156 (17.9)	< 0.001^##^
Acute kidney disease		3 (1.2)	13 (1.5)	0.77^###^
Laboratory investigations	Hemoglobin level at admission, g/dL, mean ± SD	12.4 ± 1.5	12.3 ± 2.1	0.43^***^
	Random blood glucose at admission, mg/dL, median (IQR)	163 (132, 210)	133 (112,167)	< 0.001^**^
	Highest random blood glucose-hospital stay, mg/dL median (IQR)	269.5 (205,356)	206 (140,330)	< 0.001^**^
	HbA1C during hospital stay, mean ± SD	7.0 ± 2.8	5.9 ± 2.2	< 0.001^***^
	Highest ferritin level-hospital stay, µg/L, median (IQR)	112 (95,424)	121 (96, 443)	0.56^**^
	Highest serum IL-6 level, pg/mL, median (IQR)	6 (5,20)	6 (5,6)	0.45^**^
WHO ordinal scale^	Hospitalized, no oxygen therapy	98 (40.5)	422 (48.6)	
	Oxygen by mask or nasal prongs	122 (50.4)	322 (37.1)	0.002^##^
	Non-Invasive ventilation or high-flow oxygen	18 (7.4)	103 (11.9)	
	Intubation and mechanical ventilation	4 (1.7)	21 (2.4)	
Oxygen specifications	Requirement	*n* = *242* 144 (59.5)	*n* = *868* 446 (51.4)	0.02^##^
	Mode of oxygen supplementation	*n* = *144*	*n* = *443*	< 0.001^##^
	Face mask	116 (80.6)	271 (61.2)	
	NRBM	19 (13.2)	145 (32.7)	
	HFNC	9 (6.2)	27 (6.1)	
	Duration of oxygen supplementation in days, median (IQR)	5 (3.5, 8.5)	6 (4, 9)	0.48^**^
	Ventilatory support required	*n* = *144* 22 (15.3)	*n* = *446* 124 (27.8)	0.002^##^
	Duration of NIV requirement in days, median (IQR)	4 (3,10)	5 (3,7)	0.68^**^
	Duration of MV requirement in days, median (IQR)	6 (3.5, 9)	4 (2.5, 8)	0.46^**^
Steroid specifications	Corticosteroid received	*n* = *242* 141 (58.3)	*n* = *868* 517 (59.6)	0.72^##^
	Steroid route	*n* = *141*	*n* = *514*	
	Parental	107 (75.9)	464 (89.7)	
	Oral	18 (12.8)	45 (8.7)	< 0.001^##^
	Both	16 (11.3)	5 (1.0)	
	Type of steroid received	*n* = *141*	*n* = *517*	< 0.001^##^
	Dexamethasone	50 (35.5)	323 (62.5)	
	Methylprednisolone	80 (56.7)	175 (33.9)	
	Prednisolone	9 (6.4)	11 (2.1)	
	Hydrocortisone	2 (1.4)	8 (1.5)	0.57^##^
	Whether received steroid at	*n* = *140*	*n* = *511*	
	Home	7 (5.0)	20 (3.9)	
	Hospital	133 (95.0)	491 (96.1)	
	Cumulative dose of prednisolone equivalent, median (IQR)	400 (225, 739.2)	300 (191.4, 629.3)	0.07^**^
	Duration of steroid administration (in days), median (IQR)	6 (5, 7)	6 (5, 8)	0.67^**^
Other drugs	Tocilizumab	*n* = *242* None	*n* = *868* 6 (0.7)	0.19^###^
	Other immunosuppressants	*n* = *242* 2 (0.8)	*n* = *868* 20 (2.3)	0.15^###^

^*^All characteristics are from the admission of mucormycosis cases during the COVID-19 episode

^#^More than one was reported by patients

^##^χ^2^ test

^###^Fisher's exact test

^**^Mann Whitney U test

^***^‘t’ test

^WHO Working Group on the Clinical Characterisation and Management of COVID-19 infection. A minimal common outcome measure set for COVID-19 clinical research. Lancet Infect Dis. 2020;20:e192–7

All values are expressed in n (%) unless specified

NIV: Non-invasive ventilation; MV: mechanical ventilation; NRBM: non-rebreather mask; HFNC: high flow nasal canula

Gastrointestinal symptoms include abdominal pain, diarrhoea, vomiting, loss of appetite

Otorhinolaryngological symptoms include runny nose, sore throat, ear pain, nasal discharge

Central nervous system symptoms include altered sensorium/behaviour, giddiness, weakness of limbs

### Clinical and management profile of participants receiving home-based care after COVID-19 diagnosis

A small number of patients (n = 101) received exclusively home-based care for management of COVID -19. Of them, ninety-four patients developed mucormycosis later while seven did not. Of the 94 participants who developed mucormycosis later, only 15 (15.9%) required oxygen via oxygen cylinder for a median (IQR) duration of 5 days (4, 7). None of the participants used oxygen concentrators at home. The controls treated at home did not require oxygen and did not receive steroid during their course of illness, while 23 (24.5%) mucormycosis cases received either methylprednisolone (n = 12; 52.2%) or dexamethasone (n = 11; 47.8%) for management of COVID -19 at home. It was also observed that higher proportion of patients hospitalized for management of COVID -19 developed CAM as compared to those treated at home (p < 0.001).

### Multivariable model

In multivariable analysis, adjusting for age, gender, occupation, education, residence type and various treatment related issues, five factors were identified to be independently associated with CAM (Table 3). These included history of working in dusty environments, duration of hospital stay during COVID-19 illness, presence of DM, mode of oxygen supplementation and receipt of methylprednisolone. While the odds of occurrence of CAM was about three times in individuals working in dusty environments or those who had received methylprednisolone, it was higher at 32 for those who had DM. Longer duration of hospital-stay for COVID-19 management had marginal effect on increasing the odds of developing CAM. Use of non-rebreather mask (NRBM) for oxygen supplementation was protective against development of CAM as compared to the use of face masks (Table 3).

**Table 3:** Logistic regression analysis towards identification of risk factors associated with CAM cases in India

Characteristics	OR	95% CI of OR	p value
Age in years			
< 44	Ref		
45–59	1.01	0.46, 2.26	0.96
> 60	0.63	0.28, 1.43	0.27
Sex			
Female	Ref		
Male	0.83	0.36, 1.88	0.65
History of working in dusty environment			
None	Ref		
Farming/gardening	3.23	1.34, 7.82	< 0.01
Construction sites	0.60	0.14, 2.53	0.49
Occupation			
Unemployed	Ref		
Employed	1.53	0.63, 3.71	0.34
Education			
Illiterate (no school education)	Ref		
Till 12th standard	0.78	0.29, 2.09	0.62
Graduate	1.17	0.37, 3.68	0.79
Post-graduate and above	2.19	0.43, 11.19	0.34
Type of house			
Cemented house	Ref		
Cemented house with thatched roof	0.48	0.15, 1.58	0.23
Cemented house with asbestos roof	1.17	0.30, 4.57	0.81
Mud house with thatched roof	3.77	0.77, 18.38	0.10
Duration of hospital stay in days during COVID-19	1.06	1.02, 1.11	< 0.01
Diabetes mellitus			
No	Ref		
Yes	31.83	13.96, 72.63	< 0.001
Type of oxygen supplementation			
Face mask	Ref		
NRBM	0.18	0.08, 0.41	< 0.001
HFNC	0.71	0.17, 2.95	0.64
Ventilatory support			
No	Ref		
Yes	0.51	0.22, 1.20	0.12
Type of steroid			
Dexamethasone	Ref		
Methylprednisolone	2.71	1.37, 5.37	< 0.01
Prednisolone	6.83	0.91, 51.52	0.06
Hydrocortisone	3.39	0.37, 31.36	0.28
Route of steroid			
Parenteral	Ref		
Oral	2.34	0.76, 7.21	0.14

OR: odds ratio; CI: confidence interval, NRBM: non-rebreather mask; HFNC: high flow nasal canula

Pseudo R^2^: 0.4212, n = 458

## Discussion

The present multi-site, nationwide study clearly depicted that the trend of CAM cases in hospitals in India peaked during the month of May 2021, about a month following the peak in admission of COVID-19 cases in April of the same year. This could probably be explained by complex immune response due to SARS-CoV-2 infection leading to a stage of immunosuppression after the initial cytokine storm, particularly during the second week of infection [16, 17]. Immunosuppression due to COVID-19 associated treatment with steroid could have further compounded this phenomenon. Noticeably, a similar occurrence of satellite epidemic of herpes zoster was recorded in the early 1990s following HIV epidemic induced immunosuppression among young injection heroin users in the north-eastern state of Manipur bordering Myanmar [18].

The socio-demographic profile of CAM cases in our study were similar to that in other Indian studies from Chandigarh, Delhi and Pune, and an online Mycotic Infections in COVID-19 (MUNCO) registry [8, 19–22]. Saprophytic fungi such as *Mucorales* are found in ecological spaces such as soil, dust and decomposing vegetation [23]. The results of the current study corroborated with this fact as more than one-fourth of the CAM cases (27.9%) reported dusty working environments such as either farming or gardening or both, whereas frequency of such occupational exposure among controls was only 14.7%.

Genomic sequencing of cases and controls showed a comparable presence of SARS-CoV-2 variants and did not reveal any specific association of mutation in either group. Variants of concern (Alpha, Delta and Delta derivates) or variants under investigation did not show any preferential distribution among CAM cases. However, the small sample size used in genomic sequencing precludes us from drawing any further inference from the same.

Uncontrolled DM is a known risk factor for mucormycosis. A systematic review of CAM cases globally, revealed diabetes to be a more frequently reported association from India than elsewhere (66.1% vs. 54.8%) [24]. DM as a risk factor has been reported consistently from studies conducted across India during COVID-19 pandemic [5, 19, 21, 22, 25]. There is emerging evidence of hyperglycemia induced increase in surface glucose-regulated protein (GRP78) expression on the endothelium, which in turn not only facilitates SARS CoV-2 entry by forming a complex with spike protein and angiotensin converting enzyme 2 (ACE2) receptors, but also mediates interaction with *Mucorales* spores through spore protein homologue CotH3 and promotes endothelial invasion [26, 27]. It is therefore important to monitor blood glucose levels during COVID-19 management and attain good glycemic control, with special attention on severe cases of COVID-19 who are on systemic corticosteroids.

Evidence is equivocal with respect to the role of supplemental oxygen during COVID-19 illness and subsequent development of CAM. While the current study and other studies on CAM showed higher requirement of oxygen among CAM cases during COVID illness [8, 28], studies conducted in other Indian cities such as Delhi and Pune did not witness such association [19, 21]. Interestingly, use of NRBM was found to be a protective factor against development of CAM in the current investigation. Use of high flow oxygen devices was reported to be lower in CAM group in study conducted in Delhi, India [19]. Moreover, unhygienic ways to deliver oxygen or prolonged use of same mask for more than two patients has been hypothesized for occurrence of mucormycosis [3].

Irrational corticosteroid use has been associated with development of many opportunistic infections including mucormycosis. Steroids induce immunosuppression by inhibiting macrophages and neutrophils and raise blood sugar levels, thereby increasing the risk of CAM. Inappropriate use of steroids during the second wave of COVID-19 pandemic could have resulted in prolonged hyperglycemia among pre-diabetic and diabetic patients, which in turn probably resulted in invasive mucormycosis. Corticosteroid use, one of the proven predisposing factors for mucormycosis, has not been recognized as an independent risk factor in the current study [29]. However, we observed that the odds of developing CAM were nearly three times higher with the use of methylprednisolone as compared to dexamethasone. Though efficacy of both dexamethasone and methylprednisolone is comparable, preclinical studies have demonstrated a higher lung to plasma ratio for methylprednisolone compared with dexamethasone [30]. We may hypothesize that this led to more severe immunosuppression, rendering the lung tissue incapable of getting rid of the fungal spores.

### Strengths and limitations of the study

The current investigation was a large, multi-site study conducted across four regions of the country. Validated methods were used to ascertain cases and controls. However, the current study had a few limitations as well. The information related to laboratory parameters and treatment was retrieved from paper-based medical records, which were not uniformly available across all institutes, hence not included in the multivariate model. Due to the nature of data collection (i.e., telephonically for controls), detailed information on dusty environments, use of alternative medicines or over-the-counter purchase and usage of steroids could not be explored in the current investigation. Moreover, recall bias cannot be ruled out regarding information obtained from controls. Lastly, we did not explore the impact of climate of a place and hospital environment related factors (e.g., humidifiers in the ICUs) which could serve as a potential source for mucormycosis outbreaks [31, 32]. However, this objective was beyond the scope of our investigation.

To conclude, CAM was found to be strongly associated with host factors such as diabetes mellitus and environmental factors such as working in dusty environment. Factors related to clinical management such as duration of hospital stay during COVID -19 illness and use of steroids increased the odds of CAM. On the other hand, oxygen supplementation through NRBM had a protective effect. Appropriate management of hyperglycemia, judicious use of steroids and use of NRBM during oxygen supplementation among COVID-19 patients thus emerged as potential intervention areas to prevent subsequent occurrence of mucormycosis.

## Supplementary Information

**Additional file 1.** **Figure S1:** Geographic distribution of CAM cases (n=336) and COVID-19 controls (n = 875)**. **The values for CAM cases and COVID-19 controls are representative of each of the four zones i.e., North, East, West and South plus Central.

**Additional file 2.** **Figure S2:** Symptom frequency of CAM cases (n = 336).

**Additional file 3.** **Table S1:** Details of the genomic reads mapped, total reads, pangolin lineage and the accession numbers for each of the strains retrieved.

**Additional file 4.** **Figure S3:** Single nucleotide variation (SNV) of three SARS-CoV-2 strains***. ***Single nucleotide variation of strain in the spike protein region. The X-axis shows the nucleotide mutation at the specified gene location and the Y-axis depicts the case analyzed (C: COVID-19; and M: COVID-19 infected with mucormycosis). The amino acid mutations caused due to the SNVs is shown at the top of the x- axis. The frequency of reads observed for the specific SNV is depicted using different color (maximum: red; minimum: green;) color. Dashed line indicates the segregation of cases.

## Data Availability

The next generation sequencing data generated during the current study are available in the GISAID database, (GISAID-Initiative https://www.gisaid.org/).
