# Supplementary figures and images for: A case control investigation of COVID-19 associated mucormycosis in India
Source: BMC Infect Dis. 2022 Nov 16;22:856. doi: 10.1186/s12879-022-07844-y (PMC9667849; doi:10.1186/s12879-022-07844-y)

## Slide 1
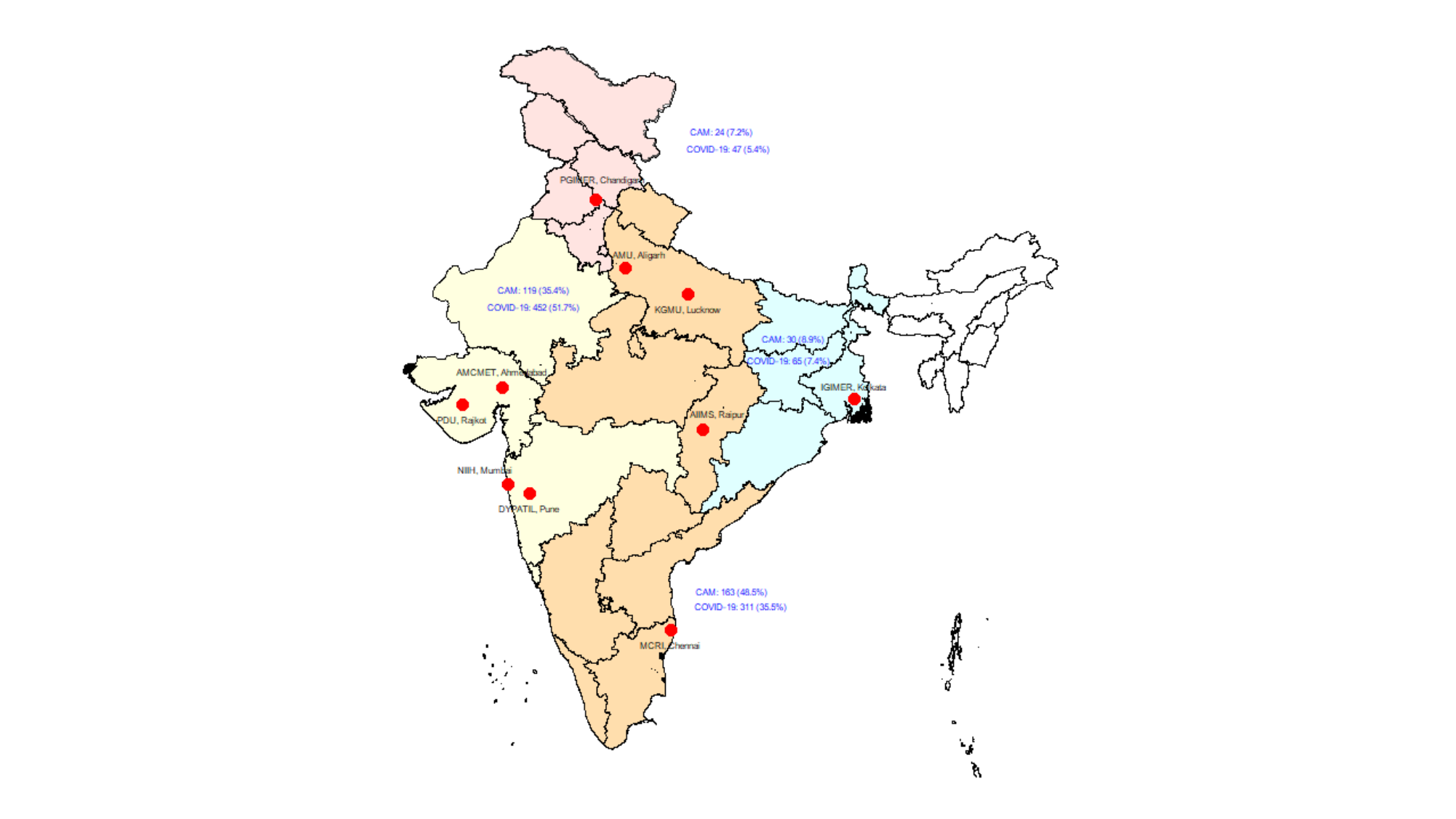

Supplement: Supplementary file 1 — Additional file 1. Figure S1: Geographic distribution of CAM cases (n=336) and COVID-19 controls (n = 875)**. **The values for CAM cases and COVID-19 controls are representative of each of the four zones i.e., North, East, West and South plus Central. [file 12879_2022_7844_MOESM1_ESM.pptx]

## Slide 1
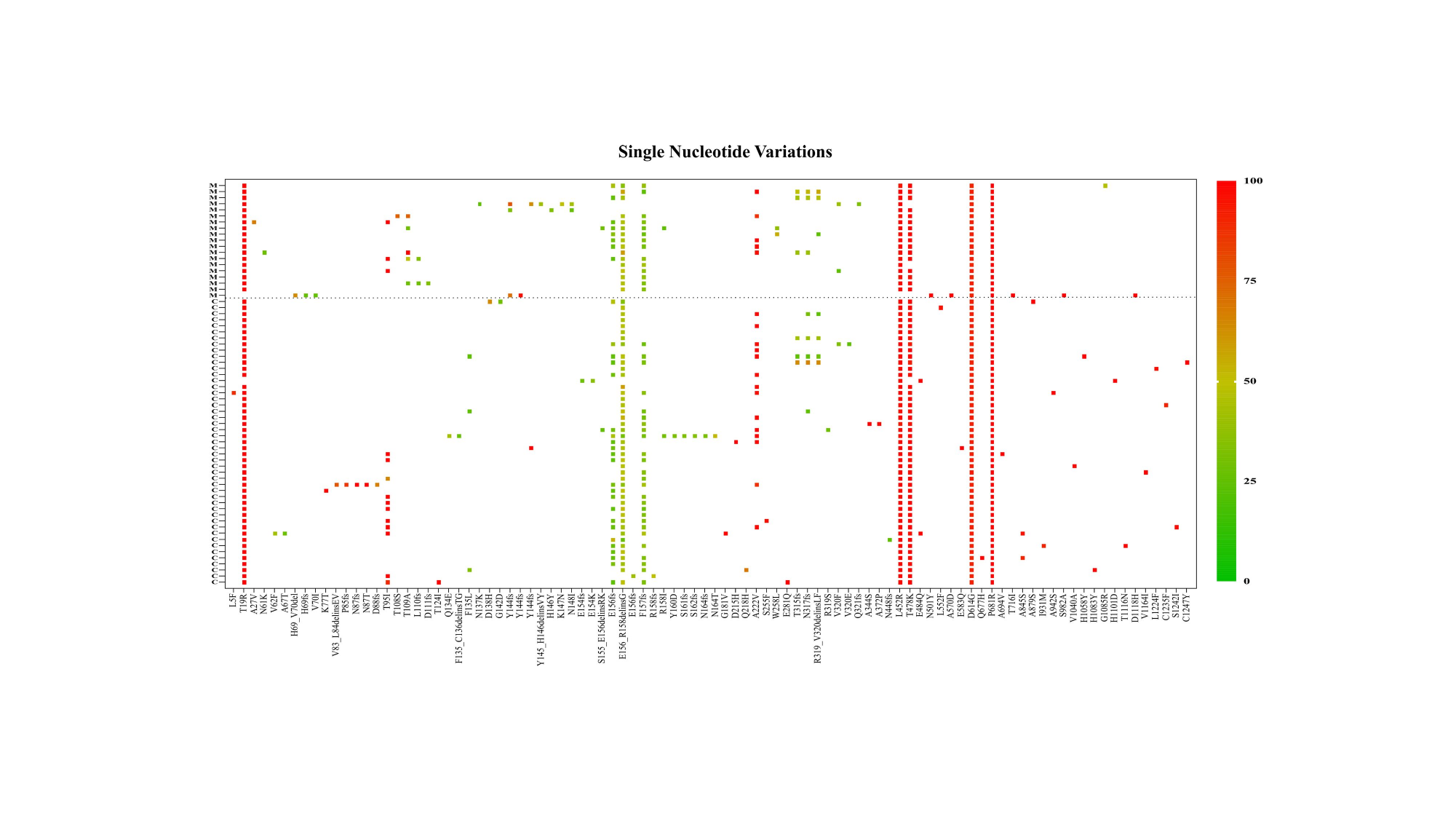

Supplement: Supplementary file 2 — Additional file 2. Figure S2: Symptom frequency of CAM cases (n = 336). [file 12879_2022_7844_MOESM2_ESM.pptx]
